# GGPP-Mediated Protein Geranylgeranylation in Oocyte Is Essential for the Establishment of Oocyte-Granulosa Cell Communication and Primary-Secondary Follicle Transition in Mouse Ovary

**DOI:** 10.1371/journal.pgen.1006535

**Published:** 2017-01-10

**Authors:** Chen Jiang, Fan Diao, Yong-Juan Sang, Na Xu, Rui-Lou Zhu, Xiu-Xing Wang, Zhong Chen, Wei-Wei Tao, Bing Yao, Hai-Xiang Sun, Xing-Xu Huang, Bin Xue, Chao-Jun Li

**Affiliations:** 1 MOE Key Laboratory of Model Animal for Disease Study, Model Animal Research Center and School of Medicine, Nanjing University, National Resource Centre for Mutant Mice, Nanjing, China; 2 Collaborative Innovation Platform for Reproductive Biology and Technology of the Medical School of Nanjing University, Nanjing, China; 3 School of Life Science and Technology, Shanghai Tech University, Shanghai, China; Syracuse University, UNITED STATES

## Abstract

Folliculogenesis is a progressive and highly regulated process, which is essential to provide ova for later reproductive life, requires the bidirectional communication between the oocyte and granulosa cells. This physical connection-mediated communication conveys not only the signals from the oocyte to granulosa cells that regulate their proliferation but also metabolites from the granulosa cells to the oocyte for biosynthesis. However, the underlying mechanism of establishing this communication is largely unknown. Here, we report that oocyte geranylgeranyl diphosphate (GGPP), a metabolic intermediate involved in protein geranylgeranylation, is required to establish the oocyte-granulosa cell communication. GGPP and geranylgeranyl diphosphate synthase (Ggpps) levels in oocytes increased during early follicular development. The selective depletion of GGPP in mouse oocytes impaired the proliferation of granulosa cells, primary-secondary follicle transition and female fertility. Mechanistically, GGPP depletion inhibited Rho GTPase geranylgeranylation and its GTPase activity, which was responsible for the accumulation of cell junction proteins in the oocyte cytoplasm and the failure to maintain physical connection between oocyte and granulosa cells. GGPP ablation also blocked Rab27a geranylgeranylation, which might account for the impaired secretion of oocyte materials such as Gdf9. Moreover, GGPP administration restored the defects in oocyte-granulosa cell contact, granulosa cell proliferation and primary-secondary follicle transition in Ggpps depletion mice. Our study provides the evidence that GGPP-mediated protein geranylgeranylation contributes to the establishment of oocyte-granulosa cell communication and then regulates the primary-secondary follicle transition, a key phase of folliculogenesis essential for female reproductive function.

## Author Summary

Folliculogenesis is a progressive and highly regulated process that requires the tight coordination of metabolism and bidirectional communication between the oocyte and granulosa cells. How this communication is established remains unclear. Here, we find that GGPP-mediated protein geranylgeranylation, a post-translational modification, is essential for the oocyte-granulosa cell communication. GGPP depletion in oocytes inhibits Rho GTPase geranylgeranylation-regulated cell adhesion and impairs Rab GTPase geranylgeranylation-directed cell secretion, which are responsible for the failure to maintain oocyte-granulosa cell communication. This communication defect is probably not able to support the proliferation of granulosa cells from one layer to multiple layers and ultimately results in the failure of the primary-secondary follicle transition and female subfertility. Our findings provide the evidence of GGPP-mediated protein geranylgeranylation involving in regulating primary-secondary follicle transition and establish a novel link between folliculogenesis and GGPP-regulated membrane dynamics.

## Introduction

Folliculogenesis is orchestrated by a complex series of cellular and molecular interactions that are evoked by the autocrine, paracrine and endocrine functions of ovarian growth factors, chemokines and steroids[1,2]. The smaller primordial and primary follicles are abundant in the ovarian cortex, where the hypoxic environment keeps them at a low metabolic rate due to insufficient vascularization and nutrition supplementation. Compared with primordial and primary follicles, the secondary follicles are found in the region closer to the ovarian medulla, where the higher O_2_ levels facilitate rapid growth and high metabolic rates[3,4]. The primary-secondary follicle transition, which is independent of the hypothalamic-pituitary-ovarian axis, is characterized by the proliferation of granulosa cells from single monolayer to multiple layers and the rapid expansion in oocyte size[5]. Indeed, this process and the subsequent oocyte development process are dependent on their bidirectional signal and material communication between the oocyte and granulosa cells[6–9].

The bidirectional communication between the oocyte and granulosa cells conveys signals from the oocyte to granulosa cells that regulate granulosa cell proliferation, including growth differentiation factor-9 (Gdf9) and bone morphogenetic protein-15 (Bmp15)[10,11]. In addition, this communication involves the transport of metabolites for biosynthesis, such as amino acids and pyruvate, from the granulosa cells to the oocyte[12]. During early follicular development, oocytes begin to express abundant cell-cell communication proteins and receptors as well as G-protein coupled receptors[13,14]. The junctional proteins expressed during follicular development include connexin 37 (gap junction protein alpha 4, Gja4), connexin 43 (gap junction protein alpha 1, Gja1), N-cadherin (cadherin 2, Cdh2), E-cadherin(cadherin 1, Cdh1), which are required to establish the bidirectional communication between oocytes and granulosa cells[15,16]. Connexin 37 localizes to the cell surface of the oocyte and provides the structural basis for the gap junctions between the oocyte and granulosa cells[17]. The loss of connexin 37 blocks oocyte growth and arrests folliculogenesis at the early antral stage[18]. Therefore, establishing communication between oocyte and granulosa cells is critical for early folliculogenesis, but the underlying mechanism remains unclear.

We found that the levels of geranylgeranyl diphosphate (GGPP), the substrate of protein geranylgeranylation, and geranylgeranyl diphosphate synthase (Ggpps) increase in oocytes during early follicular development. GGPP, a metabolic intermediate of the mevalonate pathway, is synthesized from farnesyl diphosphate (FPP) by Ggpps[19]. Both FPP and GGPP are used for the prenylation (farnesylation or geranylgeranylation) of proteins, a post-translational modification that is required for the membrane localization and activation of small GTPases such as Ras and Rho[20,21]. The activation of Rho-family GTPases is critical for the regulation of cell junctions[22]. We recently reported that Ggpps depletion in Sertoli cells enhanced Ras farnesylation and blocked spermatogenesis[23]. Thus, we speculated that GGPP and protein geranylgeranylation in the oocyte might also be essential for events that occur during early folliculogenesis, such as the primary-secondary follicle transition, due to its role in prenylating proteins associated with the regulation of cell junctions.

In this study, we generated oocyte-specific *Ggpps* knockout mice by crossing *Ggpps*^fl/fl^ mice with Ddx4-Cre transgenic mice and identified a novel function of GGPP-mediated protein geranylgeranylation in the oocyte during the primary-secondary follicle transition. Oocyte GGPP is required for Rho GTPase-regulated physical connection between oocyte and granulosa cells and Rab GTPase-directed secretion of oocyte materials such as Gdf9 and thereby has a profound impact on both the oocyte-granulosa cell communication and ovarian primary-secondary follicle transition.

## Results

### Ggpps is primarily expressed in the oocyte and associated with early follicular development

**Fig 1:**
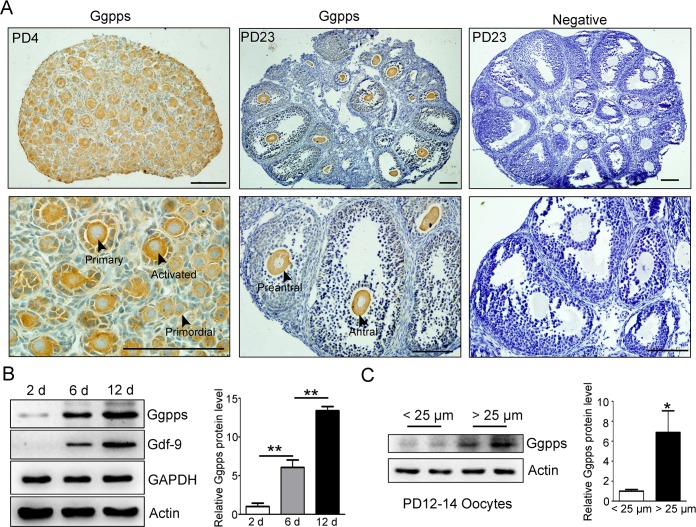
Ggpps is primarily expressed in the oocyte and associated with early follicular development. (A) Immunohistochemistry assays of Ggpps expression in ovary sections from PD 4 (primordial and primary follicle) and PD 23 (preantral and antral follicle) mice. Negative control involved exception of the primary antibody stage. Arrowheads indicated different stages of follicles. Scale bar, 100 μm. (B) Western blot analysis of ovaries from PD 2 (indicating primordial follicles), PD 6 (indicating primary follicles) and PD 12 (indicating secondary follicles) mice. Actin and GAPDH were used as internal loading controls. (C) Western blot analysis of Ggpps protein levels in the oocytes that were larger or smaller than 25 μm, respectively. Actin was used as internal loading controls. Data were presented as the mean ± SEM. * p<0.05, **p<0.01.

Immunohistochemistry assays demonstrated that Ggpps was primarily expressed in the cytoplasm of the oocyte at all of the follicular developmental stages, including the primordial follicle, primary follicle, preantral follicle, and antral follicle stages (Fig 1A). Based on the process of follicular development, we evaluated ovaries at postnatal day 2 (PD 2), PD 6, and PD 12 because these time points correspond to the development of the primordial, primary and secondary follicles, respectively[14]. Western blotting indicated that the Ggpps protein level was elevated with follicular development and that more Ggpps was found in the PD 12 ovaries (Fig 1B). To measure the Ggpps protein level in oocytes, we isolated and separated oocytes into two groups that were larger or smaller than 25 μm from the PD 12–14 ovaries. We found the protein level of Ggpps significantly increased in the oocytes that were larger than 25 μm (Fig 1C). These results suggest that GGPP-mediated protein geranylgeranylation might participate in the events that occur during early follicular development, such as primordial activation or the primary-secondary follicle transition.

### Oocyte-specific GGPP depletion impairs follicular development and female fertility

**Fig 2:**
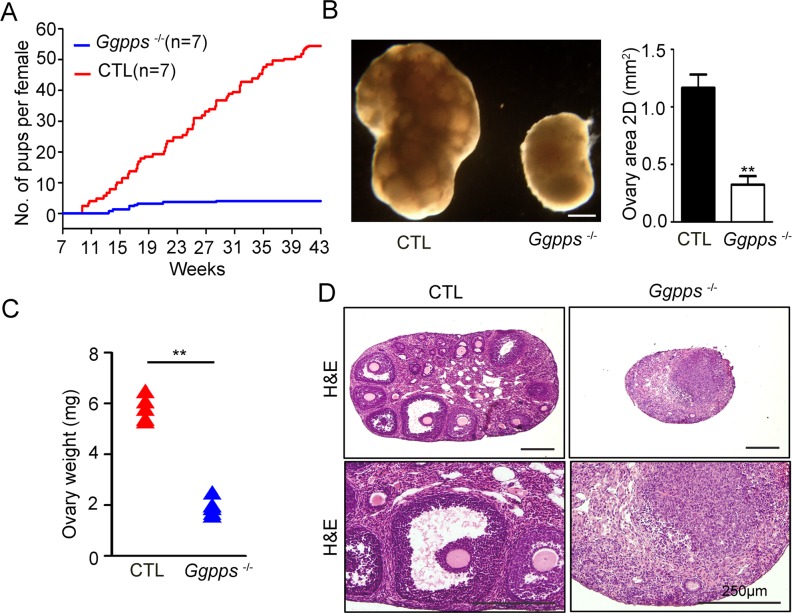
Oocyte-specific GGPP depletion impairs follicular development and female fertility. (A) A comparison of the cumulative number of pups generated by female *Ggpps*^fl/fl^ Ddx4-Cre and CTL mice. (B and C) The morphology, size and weight of 6-week-old ovaries in *Ggpps*^fl/fl^ Ddx4-Cre and CTL mice. Scale bar, 250 μm. (D) H&E staining of 6-week-old ovaries. Scale bar, 250 μm. Data were presented as the mean ± SEM. **p<0.01.

To examine the function of GGPP-mediated protein geranylgeranylation *in vivo*, we generated oocyte-specific *Ggpps* knockout mice. *Ggpps*^fl/fl^ mice were crossed with Ddx4-Cre transgenic mice to generate offsprings in which *Ggpps* was deleted during the embryonic stage. The knockout efficiency was confirmed by measuring the mRNA and protein levels of Ggpps, which showed that *Ggpps* had been efficiently deleted in oocytes (S1 Fig). To assess the fertility of these mice, we crossed female *Ggpps*^fl/fl^, Ddx4-Cre mice and control mice with C57BL/6J males between 6 and 42 weeks of age. Among 7 female *Ggpps*^fl/fl^, Ddx4-Cre mice, 4 mice revealed complete female infertility. The rest of *Ggpps*^fl/fl^, Ddx4-Cre females showed a delayed first litter (day 78.7 ± 3.9 vs. 104.7 ± 8.7), reduced litter numbers (8.3 ± 0.4 vs. 2.3 ± 0.7) and the reduced litter size (6.6 ± 0.4 vs. 3.8 ± 0.6), and they eventually became infertile after 28 weeks (Fig 2A). The dissection of 6-week-old mice indicated that both the size and weight of the ovaries in *Ggpps*^fl/fl^, Ddx4-Cre mice were significantly decreased (Fig 2B and 2C). Histological analysis of the ovaries from 6-week-old mice revealed that nearly all of the follicles had disappeared in *Ggpps*^fl/fl^, Ddx4-Cre mice (Fig 2D). These results demonstrate that oocyte-specific GGPP depletion results in loss of ovarian follicles and female subfertility.

### GGPP depletion in oocytes inhibits ovarian primary-secondary follicle transition

**Fig 3:**
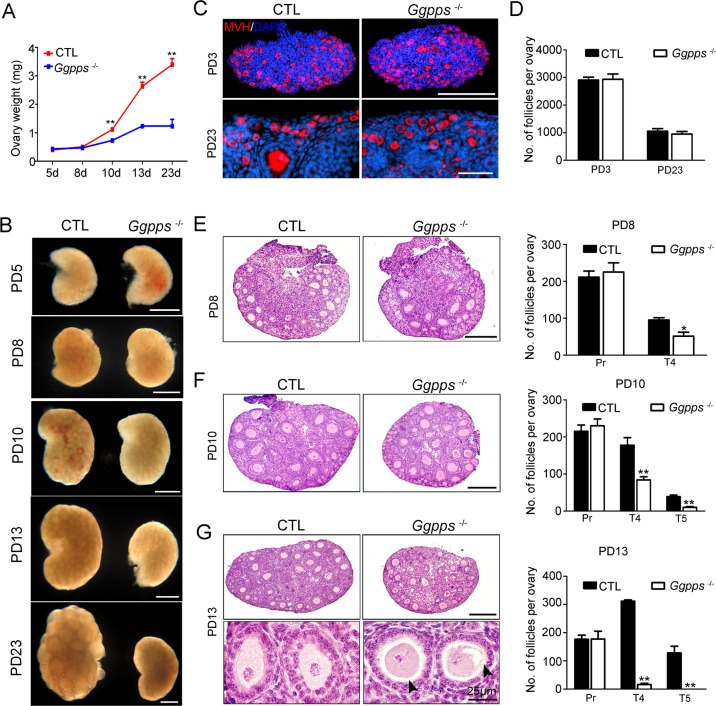
GGPP depletion in oocytes inhibits ovarian primary-secondary follicle transition. (A) Ovary weight at the indicated time points (n = 3–6). (B) The images of the ovaries at different time points were captured using a light microscope. (C and D) MVH immunofluorescence and primordial follicle numbers (n = 4) in PD 3 and PD 23 ovaries from *Ggpps*^fl/fl^ Ddx4-Cre and CTL mice. DAPI (blue) indicates the cell nuclei. (E-G) H&E staining of ovaries at the indicated time points and quantification of the different types of follicles observed in ovaries from PD 8, PD 10, and PD 13 mice, including primordial (Pri), primary (Pr), type 4 (T4), and type 5 (T5) follicles. The number of follicles per ovary was quantified as described in Materials and Methods (n = 4). Arrowheads indicate abnormal contact between the oocyte and granulosa cells. Data were presented as the mean ± SEM. * p<0.05, **p<0.01. Scale bar, 200 μm.

To determine when the follicular development was blocked, we examined the ovary weight and size of *Ggpps*^fl/fl^, Ddx4-Cre and control mice at PD 5, 8, 10, 13, and 23. We found that there were significant differences in ovarian weight and size as early as PD 10 between *Ggpps*^fl/fl^, Ddx4-Cre mice and control mice (Fig 3A and 3B). Because the expression of Ddx4-Cre starts at embryonic day E15-18[24], we first analyzed the primordial follicle abundance after GGPP depletion by using germ cell marker MVH immunostaining. The results showed that *Ggpps*^fl/fl^, Ddx4-Cre ovaries contained similar numbers of primordial follicles to those of controls both before primordial follicle activation (at PD 3) and after several rounds of activation (at PD 23) (Fig 3C and 3D), which indicates that GGPP depletion did not affect the formation or activation of primordial follicles.

However, H&E staining showed that few secondary (type 4) follicles with two layers of granulosa cells were present in *Ggpps*^fl/fl^ Ddx4-Cre ovaries, whereas the number of primary follicles (with a single layer of granulosa cells) was similar in PD 8 (Fig 3E), PD 10 (Fig 3F) and PD 13 (Fig 3G) ovaries. In contrast to the follicles that had developed into type 5 follicles (with 3–5 layers of granulosa cells) in control ovaries at PD 10 (Fig 3F) and PD 13 (Fig 3G), the follicles in knockout ovaries rarely passed the primary-secondary transition, and the majority of the activated follicles were primary follicles with a single layer of granulosa cells. To investigate the fate of those “arrested” primary follicles, we first examined apoptosis via TUNEL staining (S2 Fig). However, there were no significant differences between control and *Ggpps*^fl/fl^ Ddx4-Cre mice. Notably, the accumulation of LC3-positive puncta sharply increased in the cytoplasm of oocyte at primary follicles in *Ggpps*^fl/fl^ Ddx4-Cre mice (S3 Fig). These results indicate that GGPP depletion in oocytes blocks the primary-secondary follicle transition and those “arrested” oocytes in primary follicles undergo autophagy.

### GGPP depletion in oocytes arrests granulosa cell proliferation by impairing secretion of oocyte factors

**Fig 4:**
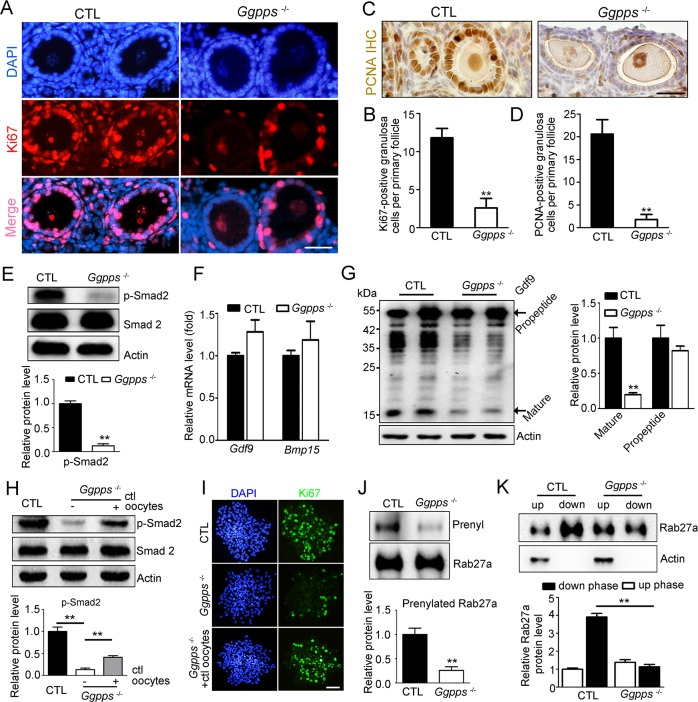
GGPP depletion in oocytes arrests granulosa cell proliferation by impairing secretion of oocyte factors. (A and B) Ki67 immunofluorescence assays and the quantification of Ki67-positive granulosa cells in the ovaries of PD 13 *Ggpps*^fl/fl^, Ddx4-Cre and CTL mice. Scale bar, 25 μm. (C and D) PCNA immunohistochemistry and the quantification of PCNA-positive granulosa cells in PD 13 ovaries. Scale bar, 25 μm. The number of Ki67-positive and PCNA-positive granulosa cells per primary follicle was quantified as described in Materials and Methods (n = 4). (E) Western blot analysis of activated Smad2 in isolated granulosa cells from PD 12–14 ovaries. (F) Quantitative PCR (qPCR) analysis of *Gdf9* and *Bmp15* expression in PD 8 ovaries. (G) Western blot analysis of Gdf9 protein in PD 8 ovaries. Actin was used as internal loading controls. The control growing oocytes were co-cultured with PD 12–14 *Ggpps*^fl/fl^, Ddx4-Cre granulosa cells. After 24 h in culture, p-Smad2 and Smad2 levels in the granulosa cell lysates were evaluated using western blot (H), Ki67 immunofluorescence was measured in isolated granulosa cells (I). Scale bar, 50 μm. (J) The prenylation of Rab27a in PD 12–14 ovaries. (K) Subcellular fractionation of Rab27a in PD 12–14 ovaries was conducted using the Triton X-114 partition method. The aqueous upper phase (up) contained the water-soluble small GTPases, and the lower organic phase (down) contained the lipid-soluble small GTPases. The data were presented as the mean ± SEM. **p<0.01.

The striking characteristics of the primary-secondary follicle transition are the proliferation of granulosa cells from one layer to multiple layers and the rapid oocyte growth. The PI3K-Akt signaling pathway in oocytes, which is generally known to regulate oocyte growth and early follicular development[25,26], was unaffected in GGPP-deleted oocytes (S4 Fig). However, we found that the defective follicles were only surrounded by one layer of granulosa cells in *Ggpps*^fl/fl^, Ddx4-Cre ovaries. To characterize the granulosa cells in the retarded primary follicles, we first examined proliferation by detecting Ki67 and PCNA expression. Notably, there were few Ki67-positive (Fig 4A and 4B) and PCNA-positive (Fig 4C and 4D) granulosa cells in the primary follicles of *Ggpps*^fl/fl^, Ddx4-Cre mice compared with controls. Smad signaling plays a well-characterized role in the regulation of proliferation of granulosa cells[11]. As expected, the activation of Smad2 and Smad1/5 was significantly inhibited in GGPP-depleted mouse ovaries (Fig 4E and S5 Fig). The activation of the Smad pathway in granulosa cells requires the stimulation by members of the transforming growth factor-β (TGF-β) superfamily, including Gdf9 and Bmp15, that are secreted from oocytes[11]. The expression of *Gdf9* and *Bmp15* mRNA were unaffected in the ovaries of *Ggpps*^fl/fl^, Ddx4-Cre mice at PD 8 (Fig 4F). Interestingly, the protein level of the mature isoform of Gdf9 (17.5 kDa) decreased dramatically in the ovaries of *Ggpps*^fl/fl^, Ddx4-Cre mice compared to the control group, whereas the level of propeptide of Gdf9 (57 kDa) was unaffected (Fig 4G). In general, Gdf9 is synthesized as pro-proteins, which are cleaved into a biologically active mature form at secretion[27]. We then co-cultured granulosa cells from *Ggpps*^fl/fl^, Ddx4-Cre ovaries with denuded granulosa-free control oocytes. After 24 h, the decrease in phosphorylated Smad2 and Ki67-positive granulosa cells of GGPP-deficient ovaries were rescued by the normal oocytes (Fig 4H and 4I). These data suggest that GGPP depletion in oocytes inhibits the proliferation of the surrounding granulosa cells probably by disrupting the secretion of factors such as Gdf9, from the oocyte, thereby inducing the arrest of the primary-secondary follicle transition.

Rab GTPase-directed endocytic trafficking pathway is well known to regulate cell secretion[28]. These Rab proteins undergo geranylgeranylation via the attachment of GGPP to the C-terminus, which is required for their membrane anchoring and activation[29,30]. We have previously reported that GGPP depletion in β-cells impaired insulin secretion by inhibiting the geranylgeranylation of Rab27a[31]. It was also reported that Rab27a was highly expressed in the oocyte and Rab27a knockout mice exhibited a reduced average litter size compared with the control[32]. Here, we measured the geranylgeranylation of Rab27a in *Ggpps*^fl/fl^, Ddx4-Cre mice. As expected, the geranylgeranylation and the hydrophobic properties of Rab27a were significantly decreased in the ovaries of *Ggpps*^fl/fl^, Ddx4-Cre mice (Fig 4J and 4K). Taken together, these results indicate that GGPP depletion in oocytes inhibits geranylgeranylation of specific Rabs, such as Rab27a, which is probably responsible for oocyte material secretion, thus blocking the Smad signaling and proliferation of granulosa cells.

### GGPP depletion in oocytes inhibits the physical connection between oocyte and granulosa cells

**Fig 5:**
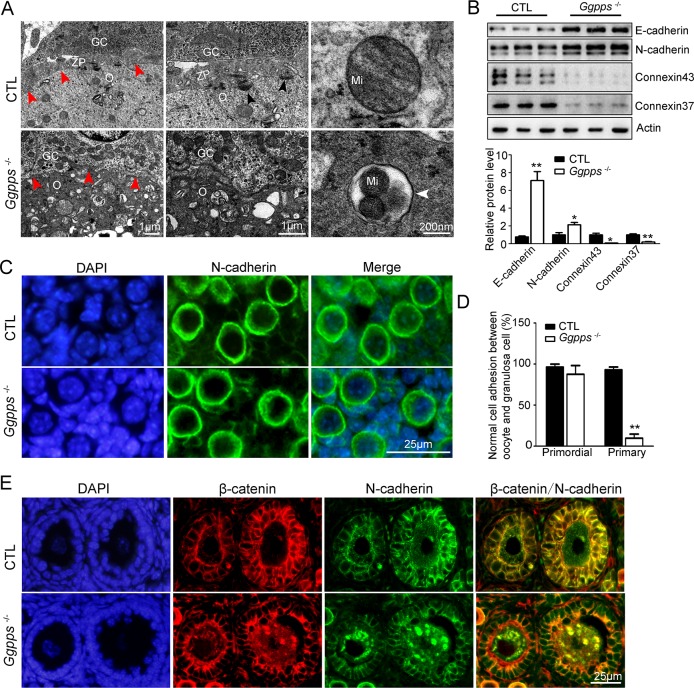
GGPP depletion in oocytes inhibits the physical connection between oocyte and granulosa cells. (A) Transmission electron microscopy analysis of PD 13 ovaries. Zona pellucida between a granulosa cell (gc) and oocyte (o) is indicated by red arrowheads in the primary follicles. Black arrowheads indicated the gap junctions. White arrowhead indicated the autophagosomes. Mi, Mitochondria. (B) Western blot analysis of N-cadherin, E-cadherin, connexin 37, and connexin 43 using lysates from PD 8 ovaries. Actin and GAPDH were used as the internal loading controls. (C-E) β-catenin, and N-cadherin immunofluorescence and the quantification of adhesion between oocyte and granulosa cells in the primordial and primary follicles of PD 13 ovaries. The cell adhesion was assessed as described in Materials and Methods (n = 4). Data were presented as the mean ± SEM. *p<0.05, **p<0.01. Scale bar, 25 μm.

It is well known that ovarian follicular development requires bidirectional communication between the oocyte and its surrounding granulosa cells[5]. Here, we found that the contact between oocyte and the granulosa cells was defective in the primary follicles of *Ggpps*^fl/fl^, Ddx4-Cre mice (Fig 3G, arrowheads). Transmission electron microscopy observation also revealed that the primary follicles showed the defective contact between oocyte and granulosa cells, as the zona pellucida (ZP, red arrowheads) and gap junction (black arrowheads) were eliminated in the primary follicles of *Ggpps*^fl/fl^, Ddx4-Cre mice (Fig 5A). Higher magnification of the images showed the ultrastructural abnormalities, such as mitophagy in the oocyte cytoplasm of primary follicle of *Ggpps*^fl/fl^, Ddx4-Cre mice (Fig 5A). In accordance with the accumulation of LC3-positive puncta in the oocyte of primary follicles in *Ggpps*^fl/fl^, Ddx4-Cre mice (S3 Fig), these results further confirmed the fate of those “arrested” primary follicles.

The protein levels of Gap junction proteins (connexin 37 and connexin 43), which are essential for oocyte-granulosa cell communication, were largely decreased in knockout ovaries as early as PD 8 (Fig 5B). Furthermore, the protein levels of E- and N- cadherin, which are essential for cell-cell adhesion, were increased in the ovaries of *Ggpps*^fl/fl^, Ddx4-Cre mice at PD 8 (Fig 5B). Further examination revealed that N-cadherin could properly localize to the oocyte membrane in primordial follicles in GGPP-depleted mice (Fig 5C and 5D). By contrast, N-cadherin and β-catenin (a component of adherens junctions binding to cadherins) accumulated in the oocyte cytoplasm but did not localize at the oocyte membrane in retarded primary follicles in *Ggpps*^fl/fl^, Ddx4-Cre mice (Fig 5D and 5E). Consistent with these data, E-cadherin, which was expressed exclusively in the oocyte membrane during follicular development, was observed in the oocyte membrane of primordial follicles but accumulated in the oocyte cytoplasm of primary follicles in knockout mice(S6 Fig). These results suggest that GGPP depletion in oocytes inhibits oocyte-granulosa cell contact in primary follicles by disturbing the E- and N- cadherin cell membrane distribution, thereby probably blocking the primary-secondary follicle transition.

### GGPP depletion disrupts cadherin-mediated cell contact by inhibiting Rho GTPase geranylgeranylation and GTPase activity

**Fig 6:**
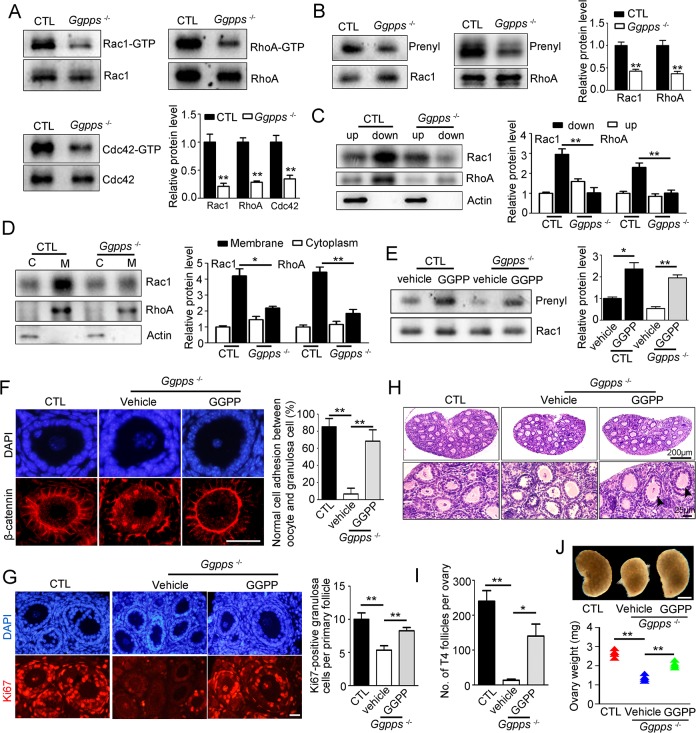
GGPP depletion disrupts cadherin-mediated cell contact by inhibiting Rho GTPase geranylgeranylation and GTPase activity. (A) The enzymatic activity of Rac1, RhoA, and Cdc42 in PD 12–14 ovaries. (B) The prenylation of Rac1 and RhoA in PD 12–14 ovaries. (C) Subcellular fractionation of Rac1 and RhoA in PD 12–14 ovaries was conducted using the Triton X-114 partition method. The aqueous upper phase contained the water-soluble small GTPases, and the lower organic phase contained the lipid-soluble small GTPases. (D) Rac1 and RhoA membrane association, as determined using ultracentrifugation, in PD 12–14 ovaries. (E) Rac1 prenylation in organ-cultured PD 12–14 ovaries by GGPP treatment (20 μM, 24 h). (F) β-catenin immunofluorescence in the primary follicles of PD 13 ovaries 5 days after daily intraperitoneal injections of GGPP (2 mg/kg). (G) Ki67 immunofluorescence, H&E staining (H), quantification of the T4 follicles (I) and ovary weight (J) in PD 13 ovaries after 5 days of daily intraperitoneal injections of GGPP (2 mg/kg). Data were presented as the mean ± SEM. *p<0.05, **p<0.01.

Rho-family GTPases play a well-characterized role in the regulation of cadherin-mediated cell-cell adhesion[22,33–35]. The enzymatic activity of Rho GTPases relies on their ability to localize properly at the membrane, which is dependent on protein geranylgeranylation, through which GGPP is transferred to its C-terminus[36]. Here, we found that the activity of Rho GTPase families, including RhoA, Rac1 and Cdc42, was sharply declined in *Ggpps*^fl/fl^, Ddx4-Cre ovaries (Fig 6A) because their geranylgeranylation was significantly inhibited (Fig 6B), which decreased their hydrophobic properties (Fig 6C) and blocked their membrane association (Fig 6D). As expected, culturing ovaries from *Ggpps*^fl/fl^, Ddx4-cre mice in medium supplemented with GGPP rescued Rac1 geranylgeranylation (Fig 6E). This rescue of GGPP was further confirmed following the intraperitoneal administration of GGPP (2 mg/kg/day) in PD 8 knockout mice. After 5 days of daily injections, cell adhesion between oocytes and granulosa cells was restored (Fig 6F). The defects in granulosa cell proliferation(Fig 6G), the number of secondary follicles (arrowheads) (Fig 6H and 6I) and ovary weight (Fig 6J) were also rescued by administering GGPP to *Ggpps*-deleted mice. Collectively, our findings indicate that GGPP-mediated protein geranylgeranylation in oocyte is required for the activation of Rho GTPases and that activated Rho GTPases regulate cadherin-mediated cell adhesion between the oocyte and granulosa cells to maintain the integrity of primary follicles required for the primary-secondary follicle transition.

**Fig 7:**
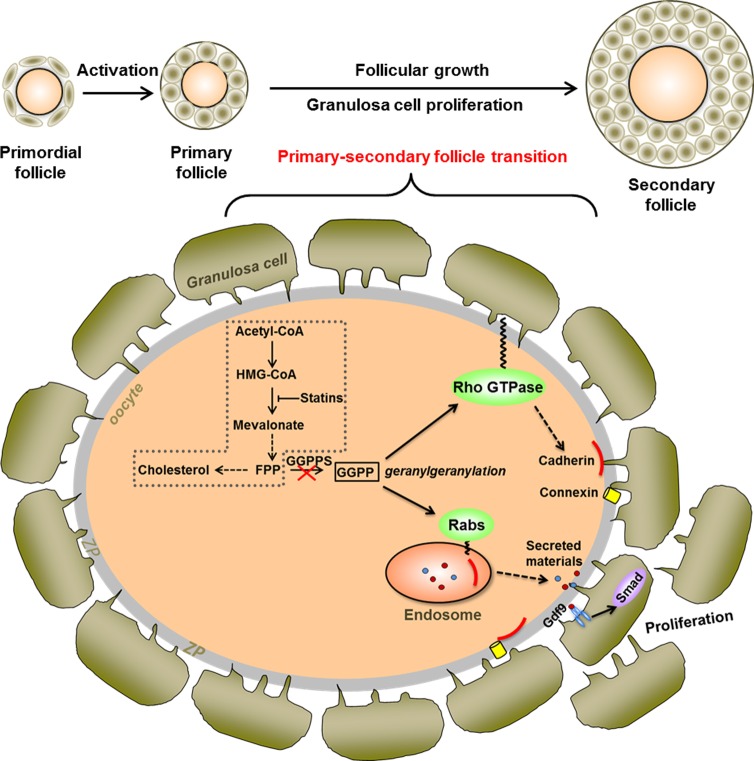
Schematic of GGPP-mediated protein geranylgeranylation involving in the regulation of primary-secondary follicle transition via remodeling oocyte-granulosa cell communication. Cholesterol was biosynthesized from farnesyl diphosphate (FPP), a metabolic intermediate of the mevalonate pathway. FPP could be catalyzed into another metabolite, geranylgeranyl diphosphate (GGPP), by geranylgeranyl diphosphate synthase (GGPPS). GGPP then was used for the geranylgeranylation and activation of Rho GTPase and Rab GTPase. The activated Rho GTPase was responsible for the localization of cell junction proteins in the oocyte membrane to maintain physical connection between oocyte and granulosa cells. The activated Rab GTPase might account for the secretion of oocyte materials such as Gdf9. The two processes were probably important for the proliferation of granulosa cells from one layer to multiple layers and ultimately promote the primary-secondary follicle transition. The pathway in the box might mainly occur in granulosa cells. Dashed arrows depicted possible mechanism of GGPP-regulated small GTPase on oocyte-granulosa cell communication.

Taken together, we report here that oocyte GGPP, which is a metabolic intermediate involved in protein geranylgeranylation, is required for Rho GTPase-regulated cell adhesion and Rab GTPase-directed secretion and thereby has a profound impact on both the oocyte-granulosa cell communication and ovarian primary-secondary follicle transition. (Fig 7).

## Discussion

After primordial follicles are activated, dormant oocytes are awakened into a phase of rapid growth, and their metabolic demands are dramatically increased[37]. During the primary-secondary follicle transition, the granulosa cells actively proliferate to form multiple layers and thereby support rapid oocyte growth[1]. Thus, the follicle must adjust to the dramatic alterations of metabolic demand that follow primordial follicle activation. In addition, gene expression profiling during early folliculogenesis showed that oocytes begun to express abundant cell-cell communication proteins (including connexin 37 and N- and E-cadherin) and receptors as well as G-protein coupled receptors[13,14]. Consistent with this, we also found that Ggpps protein and the GGPP level were associated with early folliculogenesis. The depletion of GGPP from oocytes inhibited Rho-mediated oocyte-granulosa cell contact and impaired Rab-directed cell secretion, which might be responsible for the arrest of granulosa cells proliferation and the failure of the primary-secondary follicle transition.

The bidirectional communication between oocytes and the surrounding granulosa cells relies on the localization of junction proteins to the cell membrane[15,38]. E- and N-cadherin localize to the oocyte membrane and establish oocyte-granulosa cell contacts[16,39]. Under calcium-free conditions that block cadherin-mediated cell adhesion, the contacts between oocytes and granulosa cells are maintained in primordial follicles but are lost in primary follicles[16]. Consistent with this, we also found that oocyte-granulosa cell contact was maintained in the primordial follicles of *Ggpps*^fl/fl^, Ddx4-Cre mice but was lost in primary follicles due to the accumulation of N- and E-cadherin in the oocyte cytoplasm. In connexin 37-deficient mice, folliculogenesis arrested at the early antral stage. Mouse ovarian follicles lacking connexin 43 did not develop beyond the primary follicle stage, and oocyte growth was disrupted[18,40,41]. This phenotype is similar to that of GGPP-deficient mice, and the expression of both connexin 37 and connexin 43 decreased in GGPP-deficient mice.

The enzymatic activity of Rho GTPases relies on geranylgeranylation via the attachment of GGPP to the C-terminus, which is required for their membrane anchoring and activation. We found that GGPP depletion inhibited the geranylgeranylation and activation of Rho GTPase families, which is responsible for the accumulation of N- and E-cadherin in the oocyte cytoplasm and the failure to maintain oocyte-granulosa cell contact. Here, we establish a novel link between metabolic intermediate GGPP and junction integrity through Rho GTPases. In addition, as a metabolic intermediate of the mevalonate pathway, other groups and we have reported that GGPP-mediated protein geranylgeranylation is essential for maintaining cellular homeostasis [42–45]. Therefore, identifying protein geranylgeranylation-regulated small GTPases or signaling pathways will shed a new light on the molecular mechanisms underlying oocyte development.

Intracellular vesicle formation and transport play an important role in secretion and protein turnover at the cell membrane [28,46]. It has been reported that the abnormal aggregation of membrane structures including secretory vesicles in the oocyte resulted in the failure of primary-secondary follicle transition[47]. Rab GTPases mediate the trafficking of specific cargo molecules to the cell membrane. The activity of those Rab proteins depends on GGPP-mediated geranylgeranylation and membrane anchoring[29]. It was reported that Rab27a was highly expressed in the oocyte and Rab27a knockout mice exhibited a reduced average litter size compared with the control[32]. In this study, we found the geranylgeranylation and the hydrophobic properties of Rab27a were significantly reduced in *Ggpps*^fl/fl^, Ddx4-Cre mice. In addition, we recently reported loss of GGPP in β-cells disrupted insulin secretion due to its role in Rab27a geranylgeranylation[31]. Thus, we suggested that GGPP-mediated geranylgeranylation might be essential for the material secretion and protein membrane localization of by Rab-mediated endocytic trafficking pathways.

Statins are used to lower cellular cholesterol levels in patients with hypercholesterolemia by inhibiting the rate-limiting enzyme HMG-CoA reductase of the mevalonate pathway[48,49]. Statins are also used to treat polycystic ovarian syndrome (PCOS), which is a common endocrine-metabolic disorder associated with dyslipidemia[50]. In addition to their cholesterol-lowering benefits, statins also reduce the synthesis of metabolic intermediates of the mevalonate pathway, including FPP and GGPP. As a result, prolonged statin treatment is associated with significant side effects, including myopathy and liver injury[51–53]. In this study, we found that oocyte GGPP depletion induced ovarian dysfunction and female subfertility by inhibiting protein geranylgeranylation. It was also reported that statins could inhibit blastocyst formation by preventing geranylgeranylation[54]. Therefore, the reduction of metabolic intermediates of the mevalonate pathway by statin therapy should be considered with caution.

In summary, we report that GGPP-mediated protein geranylgeranylation is essential for the membrane dynamics and the remodeling of cell communication between oocytes and granulosa cells. These findings provide the evidence of protein geranylgeranylation involving in regulating primary-secondary follicle transition and establish a novel connection between intermediate metabolites, ovarian follicular development and female fertility.

## Materials and Methods

### Ethics statement

The experimental animal facility has been accredited by the AAALAC (Association for Assessment and Accreditation of Laboratory Animal Care International), and the IACUC (Institutional Animal Care and Use Committee) of the Model Animal Research Institute of Nanjing University, who approved all animal protocols (GL12) used in this study.

### Animal studies

We generated oocyte-specific and developmental stage-specific *Ggpps* knockout mice by crossing *Ggpps*^fl/fl^ mice with Ddx4-Cre transgenic mice. The *Ggpps*^loxp/loxp^ littermates that did not express Cre and the *Ggpps*^loxp/wt^ littermates that expressed Cre were used as control mice. The knockout lines (strain 129) were backcrossed to the C57BL/6 background (the *Ggpps*-LoxP mouse background) for a minimum of 6 generations. The offspring were genotyped using PCR assays of DNA extracted from tails clippings. The reproductive capacity of the study mice was investigated by mating one C57BL/6 male with one female of the appropriate experimental strain. GGPP (Sigma) was intraperitoneally administered daily at a dose of 2 mg/kg to Ggpps^fl/fl^, Ddx4-Cre mice from PD 8 to PD 13. The dose of GGPP was determined as described in our previous study[23].

### Materials

GGPP was purchased from Sigma-Aldrich. The anti-E-cadherin (#3195p), anti-connexin43 (#3512p), anti-p-Smad2 (#3108p), anti-Smad2 (#5339p), anti-p-Smad1/5 (#9516p), anti-Smad1 (#9743), anti-p-Erk (#9106), anti-Erk (#4695), anti-p-Akt (#4060s), anti-Akt (#9272), anti-Foxo3a (#12829p), anti-p-S6 (#9206s), and anti-S6 (#2708) antibodies were purchased from Cell Signaling Technology. The anti-MVH (ab13840), anti-Ki67 (ab15580), anti-p-Foxo3a (ab47285), anti-Cdc42 (ab187643), anti-LC3B (ab48394), anti-Rab27a (ab55667), anti-connexin 37 (ab181701) and anti-Gdf9 (ab93892) were purchased from Abcam. The anti-Ggpps (sc-271679), anti-N-cadherin (sc-7939), anti-GAPDH (sc-47724), anti-Rac1 (sc-95), anti-RhoA (sc-418), and anti-PCNA (sc-25280) antibodies were purchased from Santa Cruz Biotechnology. Anti-β-catenin (610153) was purchased from BD Biosciences.

### Quantification and assessment of ovarian follicles and cell adhesion and quantification of Ki67-positive and PCNA-positive granulosa cells

The various types of ovarian follicles were quantified as previously described[25]. Briefly, the ovaries were fixed in Bouin’s fixative, dehydrated, and embedded in paraffin. The paraffin-embedded ovaries were serially sectioned into 8 μm slices and stained with hematoxylin and eosin (H&E). Ovarian follicles at different stages of development, including primordial follicles (with a single layer of flattened granulosa cells), primary follicles (with a single layer of cuboidal granulosa cells), type 4 follicles (with two layers of cuboidal granulosa cells) and type 5 follicles (with 3–5 layers of granulosa cells) were counted in every fifth section of an ovary in accordance with the well-accepted standards established by Pedersen and Peters[55]. Given that this procedure samples one-fifth of the entire ovary volume, the total number of follicles per ovary was estimated by multiplying the cumulative counts for each ovary by a correction factor of five[56]. The Ki67-positive and PCNA-positive granulosa cells were counted in twenty primary follicles of the same oocyte diameter per ovary. The normal cell adhesion between oocyte and granulosa cells was assessed by the membrane localization of cell adhesion marker (N- / E-cadherin and β-catenin). Twenty primary follicles with the same diameter of oocyte were counted. Only those follicles containing an oocyte with a clearly visible nucleus were scored. All of the counts were conducted by a single trained ovarian histologist in a blinded manner.

### Immunohistochemistry, immunofluorescence, transmission electron microscopy (TEM), and the TUNEL assay

For the immunohistochemistry and immunofluorescence analysis, the ovaries were fixed in 4% paraformaldehyde, embedded in paraffin, and sectioned into 5 μm slices. After antigen retrieval, the slides were blocked with goat serum and incubated with primary antibody [mouse anti-Ggpps (1:50), mouse anti-PCNA (1:200), rabbit anti-MVH/DDX4 (1:200), rabbit anti-N-cadherin (1:200), rabbit anti-E-cadherin (1:200), mouse anti-β-catenin (1:200), rabbit anti-LC3B or rabbit anti-Ki67 (1:200)] overnight at 4°C. An Elite ABC kit and DAB substrate was used for the immunohistochemistry analysis. Alexa Fluor 594 (Invitrogen) was used as the secondary antibody in immunofluorescence assays. The ovaries were prepared for TEM, and the TEM images were acquired as previously described[57]. Apoptosis was assessed using a florescent TUNEL kit (G3250; Promega).

### Oocyte and granulosa cell isolation

The oocytes were isolated from PD12-14 ovaries as previously described[25]. Briefly, the ovaries were homogenized and incubated in 0.05% collagenase dissolved in Dulbecco’s modified Eagle’s medium-F12 (DMEM/F12) (Invitrogen) at 37°C while being frequently agitated. After the tissue digestion, the mixture of cells (oocytes and granulosa cells) were cultured in a 10 cm tissue culture dish with DMEM/F12 medium for 12 h to allow the granulosa cells and other ovarian cells to attach to the plastic. The unattached oocytes were recovered by centrifugation at 1,000 rpm for 5 min. The oocytes were separated into two groups that were larger or smaller than 25μm, respectively, with a cell-dispersing screen with 25μm opening (millipore). The oocyte-granulosa cell co-culture experiment was performed as previously reported[58]. Briefly, oocytes and granulosa cells were surgically removed from oocyte-granulosa cells complexes of PD 12–14 ovaries after collagenase digestion. Granulosa cells were then co-cultured with growing oocytes (4 oocytes/μl) under mineral oil in 35-mm dishes in 50–100 μl culture medium for 24h.

### Prenylation and membrane association measurements

Protein prenylation was measured as described in our previous studies[23,59]. To evaluate Rac1 and RhoA membrane association, a subcellular fractionation of the ovaries was conducted using the Triton X-114 partition method and ultracentrifugation. The ovaries were lysed in lysis buffer containing protease inhibitors and subsequently centrifuged at 12,000 g for 15 min. An equal volume of 4% Triton X-114 was added to the supernatant, and the reaction was incubated at 37°C for 5 min to solubilize and fractionate the lipid-rich cell membranes. The aqueous upper phase contains enriched intracellular proteins, and the organic lower phase contains highly enriched membrane-associated proteins. For ultracentrifugation, the ovaries were lysed and homogenized with ice-cold Dounce tissue homogenizer, and the lysates were centrifuged at 100,000 x g for 30 min (4°C). The supernatant represents the cytosolic fraction, and the pellet represents the membrane fraction. All of the aforementioned samples were analyzed using western blot assays with anti-Rac1 and anti-RhoA.

### mRNA and protein expression assays

Total RNA was extracted from ovaries or cells using TRIzol reagent according to the manufacturer’s protocol. Real-time PCR was conducted using SYBR Green and an Applied Biosystems 7300 Sequence Detection System. The relative expression level values were normalized to actin to calculate fold-changes in expression. To analyze protein expression, the cells or ovaries were washed in ice-cold PBS and harvested using RIPA buffer supplemented with protease inhibitors. The resulting supernatant fraction was separated using SDS-PAGE, and the membranes were blotted with the appropriate antibodies. For the immunoprecipitation assays, antibodies against Rac1, RhoA and Rab27a were used to form immune complexes with the indicated protein in the cell lysates. The immune complexes were precipitated using protein A/G agarose beads. After several washes, the samples were boiled and analyzed using western blot with anti-prenyl antibody. The activity of Rac1, RhoA and Cdc42 was assessed using the appropriate activation Assay Kit purchased from NewEast Biosciences.

### In vitro ovary organ cultures

Ovaries were collected at PD8 and placed on 0.4 μm floating filters (Millicell-CM) in 1.1 ml of DMEM/F12 Media (Invitrogen) with 0.1% Albumax (Invitrogen), 0.1% BSA (Invitrogen), 5X ITS-X (Life Technologies), and 0.05 mg/ml L-ascorbic acid (Sigma) as previously described[60]. The medium was changed every 24 h.

### Statistics

All of the data are presented as the mean ± SEM. All of the data were analyzed using a 2-tailed Student’s t-test between 2 groups. A P value less than 0.05 was considered statistically significant. All of the statistical analyses were performed using the GraphPad Prism 5 software.

## Supporting Information

S1 Fig *Ggpps* was specifically deleted in oocytes. (A) Quantitative PCR (qPCR) analysis of *Ggpps* in PD12-14 *Ggpps*^fl/fl^ Ddx4-Cre and CTL oocytes. (B) Western blot analysis of Ggpps in PD12-14 oocytes. (C) Ggpps IHC in PD13 ovaries. Scale bar, 100 μm. Data were presented as the mean ± SEM. **p<0.01.
(TIF)

S2 Fig Apoptosis in the ovaries was assayed using TUNEL. The green dots represent apoptotic cells and DAPI (blue) indicates cell nuclei. Scale bar, 200 μm.
(TIF)

S3 Fig LC3B immunofluorescence and the quantification of oocyte autophagy in primary follicles of PD 13 ovaries. The Red dots represent LC3B and DAPI (blue) indicates cell nuclei. Scale bar, 25 μm. Data were presented as the mean ± SEM. **p<0.01.
(TIF)

S4 Fig The oocyte PI3K-Akt signaling in isolated oocytes from PD12-14 ovaries. (TIF)

S5 Fig The smad1/5 activation in isolated granulosa cells from PD12-14 ovaries. Data were presented as the mean ± SEM. **p<0.01.
(TIF)

S6 Fig E-cadherin and β-catenin immunofluorescence of PD 13 ovaries. The red represented β-catenin and the green indicates E-cadherin. Scale bar, 25 μm.
(TIF)

S1 Table Primer sequences. (PDF)

## References

[1] Richards JS, Pangas SA (2010) The ovary: basic biology and clinical implications. The Journal of clinical investigation 120: 963 10.1172/JCI41350 20364094 PMC2846061

[2] Richards JS, Russell DL, Ochsner S, Hsieh M, Doyle KH, et al (2002) Novel signaling pathways that control ovarian follicular development, ovulation, and luteinization. Recent Progress in Hormone Research 57: 195–220. 12017544 10.1210/rp.57.1.195

[3] Makanji Y, Tagler D, Pahnke J, Shea LD, Woodruff TK (2014) Hypoxia-mediated carbohydrate metabolism and transport promote early-stage murine follicle growth and survival. American Journal of Physiology-Endocrinology and Metabolism 306: E893–E903. 10.1152/ajpendo.00484.2013 24569591 PMC3989738

[4] Picton H (2001) Activation of follicle development: the primordial follicle. Theriogenology 55: 1193–1210. 11327680 10.1016/s0093-691x(01)00478-2

[5] Matzuk MM, Burns KH, Viveiros MM, Eppig JJ (2002) Intercellular communication in the mammalian ovary: oocytes carry the conversation. Science 296: 2178–2180. 10.1126/science.1071965 12077402

[6] Su Y-Q, Sugiura K, Wigglesworth K, O'Brien MJ, Affourtit JP, et al (2008) Oocyte regulation of metabolic cooperativity between mouse cumulus cells and oocytes: BMP15 and GDF9 control cholesterol biosynthesis in cumulus cells. Development 135: 111–121. 10.1242/dev.009068 18045843

[7] Wigglesworth K, Lee K-B, O’Brien MJ, Peng J, Matzuk MM, et al (2013) Bidirectional communication between oocytes and ovarian follicular somatic cells is required for meiotic arrest of mammalian oocytes. Proceedings of the National Academy of Sciences 110: E3723–E3729. 10.1073/pnas.1314829110 PMC3785791 23980176

[8] Zhang M, Su Y-Q, Sugiura K, Xia G, Eppig JJ (2010) Granulosa cell ligand NPPC and its receptor NPR2 maintain meiotic arrest in mouse oocytes. Science 330: 366–369. 10.1126/science.1193573 20947764 PMC3056542

[9] Shuhaibar LC, Egbert JR, Norris RP, Lampe PD, Nikolaev VO, et al (2015) Intercellular signaling via cyclic GMP diffusion through gap junctions restarts meiosis in mouse ovarian follicles. Proceedings of the National Academy of Sciences 112: 5527–5532. 10.1073/pnas.1423598112 PMC4418852 25775542

[10] Dong JW, Albertini DF, Nishimori K, Kumar TR, Lu NF, et al (1996) Growth differentiation factor-9 is required during early ovarian folliculogenesis. Nature 383: 531–535. 10.1038/383531a0 8849725

[11] Gilchrist RB, Lane M, Thompson JG (2008) Oocyte-secreted factors: regulators of cumulus cell function and oocyte quality. Human Reproduction Update 14: 159–177. 10.1093/humupd/dmm040 18175787

[12] Sugiura K, Eppig JJ (2005) Society for Reproductive Biology Founders' Lecture 2005. Control of metabolic cooperativity between oocytes and their companion granulosa cells by mouse oocytes. Reproduction, Fertility and Development 17: 667–674. 10.1071/rd05071 16364219

[13] Dharma SJ, Modi DN, Nandedkar TD (2009) Gene expression profiling during early folliculogenesis in the mouse ovary. Fertility and sterility 91: 2025–2036. 10.1016/j.fertnstert.2008.02.088 18504043

[14] Pan H, O'Brien MJ, Wigglesworth K, Eppig JJ, Schultz RM (2005) Transcript profiling during mouse oocyte development and the effect of gonadotropin priming and development in vitro. Developmental biology 286: 493–506. 10.1016/j.ydbio.2005.08.023 16168984

[15] Kidder GM, Mhawi AA (2002) Gap junctions and ovarian folliculogenesis. Reproduction 123: 613–620. 12006089 10.1530/rep.0.1230613

[16] Mora JM, Fenwick MA, Castle L, Baithun M, Ryder TA, et al (2012) Characterization and significance of adhesion and junction-related proteins in mouse ovarian follicles. Biology of reproduction 86: 153 10.1095/biolreprod.111.096156 22321830

[17] Veitch GI, Gittens JE, Shao Q, Laird DW, Kidder GM (2004) Selective assembly of connexin37 into heterocellular gap junctions at the oocyte/granulosa cell interface. Journal of cell science 117: 2699–2707. 10.1242/jcs.01124 15138288

[18] Simon AM, Goodenough DA, Li E, Paul DL (1997) Female infertility in mice lacking connexin 37. 10.1038/385525a0 9020357

[19] Goldstein JL, Brown MS (1990) Regulation of the mevalonate pathway. Nature 343: 425–430. 10.1038/343425a0 1967820

[20] Walker K, Olson MF (2005) Targeting Ras and Rho GTPases as opportunities for cancer therapeutics. Current opinion in genetics & development 15: 62–68. 15661535 10.1016/j.gde.2004.11.001

[21] Konstantinopoulos PA, Karamouzis MV, Papavassiliou AG (2007) Post-translational modifications and regulation of the RAS superfamily of GTPases as anticancer targets. Nature Reviews Drug Discovery 6: 541–555. 10.1038/nrd2221 17585331

[22] Fukata M, Kaibuchi K (2001) Rho-family GTPases in cadherin-mediated cell—cell adhesion. Nature Reviews Molecular Cell Biology 2: 887–897. 10.1038/35103068 11733768

[23] Wang X-X, Ying P, Diao F, Wang Q, Ye D, et al (2013) Altered protein prenylation in Sertoli cells is associated with adult infertility resulting from childhood mumps infection. The Journal of experimental medicine 210: 1559–1574. 10.1084/jem.20121806 23825187 PMC3727317

[24] Gallardo T, Shirley L, John GB, Castrillon DH (2007) Generation of a germ cell‐specific mouse transgenic Cre line, Vasa‐Cre. Genesis 45: 413–417. 10.1002/dvg.20310 17551945 PMC2597027

[25] Reddy P, Liu L, Adhikari D, Jagarlamudi K, Rajareddy S, et al (2008) Oocyte-specific deletion of Pten causes premature activation of the primordial follicle pool. Science 319: 611–613. 10.1126/science.1152257 18239123

[26] Reddy P, Zheng W, Liu K (2010) Mechanisms maintaining the dormancy and survival of mammalian primordial follicles. Trends Endocrinol Metab 21: 96–103. 10.1016/j.tem.2009.10.001 19913438

[27] Gilchrist RB, Ritter L, Cranfield M, Jeffery L, Amato F, et al (2004) Immunoneutralization of growth differentiation factor 9 reveals it partially accounts for mouse oocyte mitogenic activity. Biology of reproduction 71: 732–739. 10.1095/biolreprod.104.028852 15128595

[28] Ostrowski M, Carmo NB, Krumeich S, Fanget I, Raposo G, et al (2010) Rab27a and Rab27b control different steps of the exosome secretion pathway. Nature cell biology 12: 19–30. 10.1038/ncb2000 19966785

[29] Leung KF, Baron R, Seabra MC (2006) Thematic review series: lipid posttranslational modifications. geranylgeranylation of Rab GTPases. Journal of lipid research 47: 467–475. 10.1194/jlr.R500017-JLR200 16401880

[30] Pfeffer S, Aivazian D (2004) Targeting Rab GTPases to distinct membrane compartments. Nature Reviews Molecular Cell Biology 5: 886–896. 10.1038/nrm1500 15520808

[31] Jiang S, Shen D, Jia WJ, Han X, Shen N, et al (2016) GGPPS‐mediated Rab27A geranylgeranylation regulates β cell dysfunction during type 2 diabetes development by affecting insulin granule docked pool formation. The Journal of pathology 238: 109–119. 10.1002/path.4652 26434932

[32] Tolmachova T, Anders R, Stinchcombe J, Bossi G, Griffiths GM, et al (2004) A general role for Rab27a in secretory cells. Molecular biology of the cell 15: 332–344. 10.1091/mbc.E03-07-0452 14617806 PMC307551

[33] Watanabe T, Sato K, Kaibuchi K (2009) Cadherin-mediated intercellular adhesion and signaling cascades involving small GTPases. Cold Spring Harbor perspectives in biology 1: a003020 10.1101/cshperspect.a003020 20066109 PMC2773633

[34] Braga VM, Machesky LM, Hall A, Hotchin NA (1997) The small GTPases Rho and Rac are required for the establishment of cadherin-dependent cell–cell contacts. The Journal of cell biology 137: 1421–1431. 9182672 10.1083/jcb.137.6.1421 PMC2132529

[35] Citi S, Guerrera D, Spadaro D, Shah J (2014) Epithelial junctions and Rho family GTPases: the zonular signalosome. Small GTPases 5: e973760. 10.4161/21541248.2014.973760 PMC4601189 25483301

[36] Roberts PJ, Mitin N, Keller PJ, Chenette EJ, Madigan JP, et al (2008) Rho Family GTPase modification and dependence on CAAX motif-signaled posttranslational modification. Journal of Biological Chemistry 283: 25150–25163. 10.1074/jbc.M800882200 18614539 PMC2533093

[37] Wassarman P, Albertini D (1994) The mammalian ovum. The physiology of reproduction 1: 79–122.

[38] Winterhager E, Kidder GM (2015) Gap junction connexins in female reproductive organs: implications for women's reproductive health. Human reproduction update: dmv007. 10.1093/humupd/dmv007 25667189

[39] Wang C, Roy SK (2010) Expression of E-cadherin and N-cadherin in perinatal hamster ovary: possible involvement in primordial follicle formation and regulation by follicle-stimulating hormone. Endocrinology 151: 2319–2330. 10.1210/en.2009-1489 20219978 PMC2869259

[40] Ackert CL, Gittens JE, O'Brien MJ, Eppig JJ, Kidder GM (2001) Intercellular communication via connexin43 gap junctions is required for ovarian folliculogenesis in the mouse. Developmental biology 233: 258–270. 10.1006/dbio.2001.0216 11336494

[41] Gershon E, Plaks V, Aharon I, Galiani D, Reizel Y, et al (2008) Oocyte-directed depletion of connexin43 using the Cre-LoxP system leads to subfertility in female mice. Developmental biology 313: 1–12. 10.1016/j.ydbio.2007.08.041 18005958

[42] Sorrentino G, Ruggeri N, Specchia V, Cordenonsi M, Mano M, et al (2014) Metabolic control of YAP and TAZ by the mevalonate pathway. Nature cell biology 16: 357–366. 10.1038/ncb2936 24658687

[43] Tao W, Wu J, Xie B-X, Zhao Y-Y, Shen N, et al (2015) Lipid-induced muscle insulin resistance is mediated by GGPPS via modulation of the RhoA/Rho-kinase signaling pathway. Journal of Biological Chemistry: jbc. M115. 657742. 10.1074/jbc.M115.657742 PMC4536415 26112408

[44] Yu X, Shen N, Zhang ML, Pan FY, Wang C, et al (2011) Egr‐1 decreases adipocyte insulin sensitivity by tilting PI3K/Akt and MAPK signal balance in mice. The EMBO journal 30: 3754–3765. 10.1038/emboj.2011.277 21829168 PMC3173797

[45] Liu Y, Samuel BS, Breen PC, Ruvkun G (2014) Caenorhabditis elegans pathways that surveil and defend mitochondria. Nature 508: 406–410. 10.1038/nature13204 24695221 PMC4102179

[46] Baum B, Georgiou M (2011) Dynamics of adherens junctions in epithelial establishment, maintenance, and remodeling. The Journal of cell biology 192: 907–917. 10.1083/jcb.201009141 21422226 PMC3063136

[47] Udagawa O, Ishihara T, Maeda M, Matsunaga Y, Tsukamoto S, et al (2014) Mitochondrial fission factor Drp1 maintains oocyte quality via dynamic rearrangement of multiple organelles. Current Biology 24: 2451–2458. 10.1016/j.cub.2014.08.060 25264261

[48] Istvan ES, Deisenhofer J (2001) Structural mechanism for statin inhibition of HMG-CoA reductase. Science 292: 1160–1164. 10.1126/science.1059344 11349148

[49] Unit ES (2005) Efficacy and safety of cholesterol-lowering treatment: prospective meta-analysis of data from 90 056 participants in 14 randomised trials of statins. Lancet 366: 1267–1278. 10.1016/S0140-6736(05)67394-1 16214597

[50] Banaszewska B, Spaczyński R, Pawelczyk L (2010) [Statins in the treatment of polycystic ovary syndrome]. Ginekologia polska 81: 618–621. 20873125

[51] Russo MW, Hoofnagle JH, Gu J, Fontana RJ, Barnhart H, et al (2014) Spectrum of statin hepatotoxicity: Experience of the drug‐induced liver injury network. Hepatology 60: 679–686. 10.1002/hep.27157 24700436 PMC4110177

[52] Schirris TJ, Renkema GH, Ritschel T, Voermans NC, Bilos A, et al (2015) Statin-Induced Myopathy Is Associated with Mitochondrial Complex III Inhibition. Cell metabolism 22: 399–407. 10.1016/j.cmet.2015.08.002 26331605

[53] Armitage J (2007) The safety of statins in clinical practice. The Lancet 370: 1781–1790. 10.1016/S0140-6736(07)60716-8 17559928

[54] Alarcon VB, Marikawa Y (2016) Statins inhibit blastocyst formation by preventing geranylgeranylation. Molecular human reproduction: gaw011. 10.1093/molehr/gaw011 PMC4847613 26908642

[55] PEDERSEN T, Peters H (1968) Proposal for a classification of oocytes and follicles in the mouse ovary. Journal of reproduction and fertility 17: 555–557. 5715685 10.1530/jrf.0.0170555

[56] Johnson J, Canning J, Kaneko T, Pru JK, Tilly JL (2004) Germline stem cells and follicular renewal in the postnatal mammalian ovary. Nature 428: 145–150. 10.1038/nature02316 15014492

[57] Da Silva-Buttkus P, Jayasooriya GS, Mora JM, Mobberley M, Ryder TA, et al (2008) Effect of cell shape and packing density on granulosa cell proliferation and formation of multiple layers during early follicle development in the ovary. Journal of cell science 121: 3890–3900. 10.1242/jcs.036400 19001500

[58] Sugiura K, Pendola FL, Eppig JJ (2005) Oocyte control of metabolic cooperativity between oocytes and companion granulosa cells: energy metabolism. Developmental biology 279: 20–30. 10.1016/j.ydbio.2004.11.027 15708555

[59] Xu N, Guan S, Chen Z, Yu Y, Xie J, et al (2015) The alteration of protein prenylation induces cardiomyocyte hypertrophy through Rheb–mTORC1 signalling and leads to chronic heart failure. The Journal of pathology. 10.1002/path.4480 25385233

[60] Nilsson EE, Kezele P, Skinner MK (2002) Leukemia inhibitory factor (LIF) promotes the primordial to primary follicle transition in rat ovaries. Molecular and cellular endocrinology 188: 65–73. 11911947 10.1016/s0303-7207(01)00746-8

