# Supplementary figures and images for: GGPP-Mediated Protein Geranylgeranylation in Oocyte Is Essential for the Establishment of Oocyte-Granulosa Cell Communication and Primary-Secondary Follicle Transition in Mouse Ovary
Source: PLoS Genet. 2017 Jan 10;13(1):e1006535. doi: 10.1371/journal.pgen.1006535 (PMC5224981; doi:10.1371/journal.pgen.1006535)

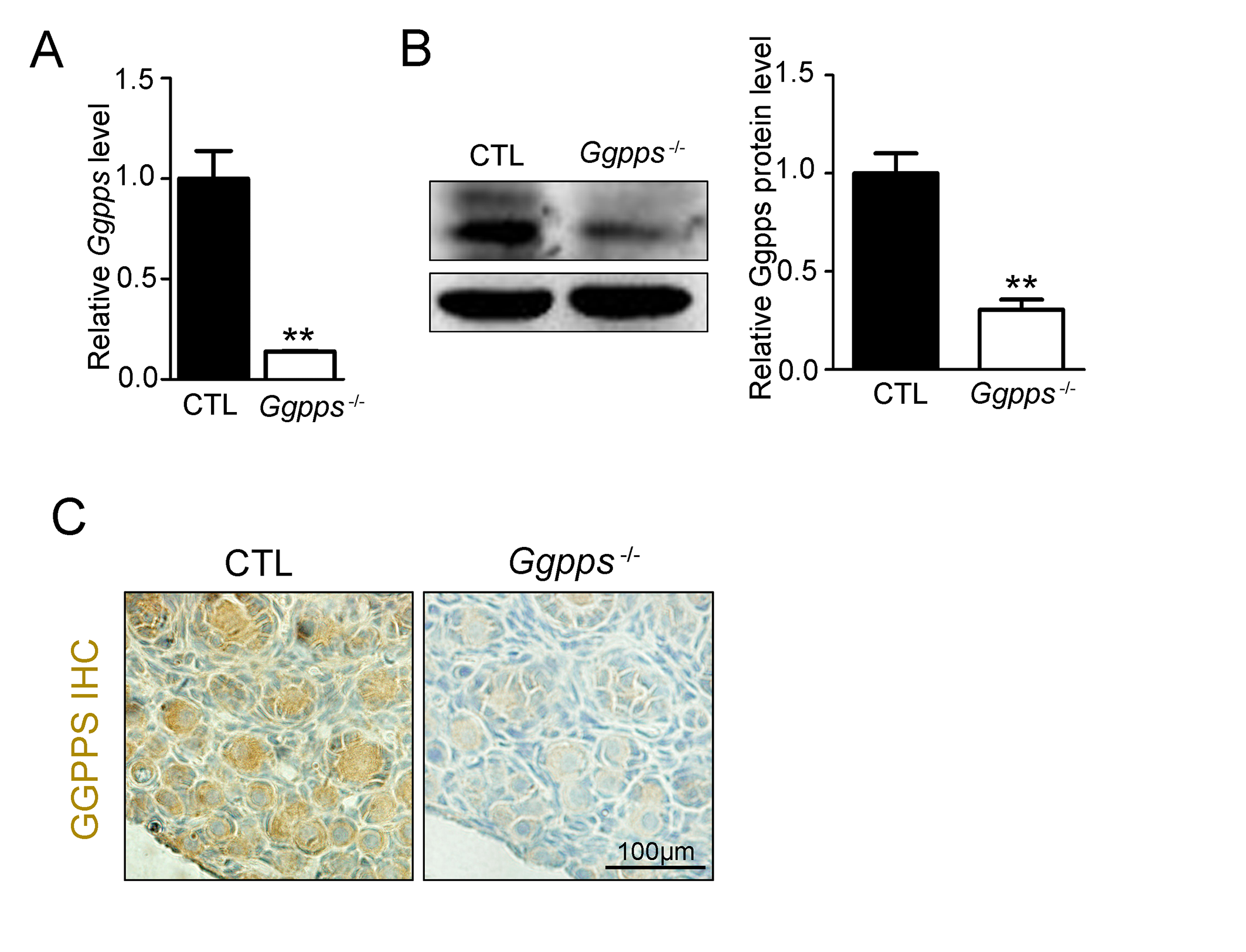

Supplement: S1 Fig — (A) Quantitative PCR (qPCR) analysis of Ggpps in PD12-14 Ggppsfl/fl Ddx4-Cre and CTL oocytes. (B) Western blot analysis of Ggpps in PD12-14 oocytes. (C) Ggpps IHC in PD13 ovaries. Scale bar, 100 μm. Data were presented as the mean ± SEM. **p<0.01. (TIF) [file pgen.1006535.s001.tif]

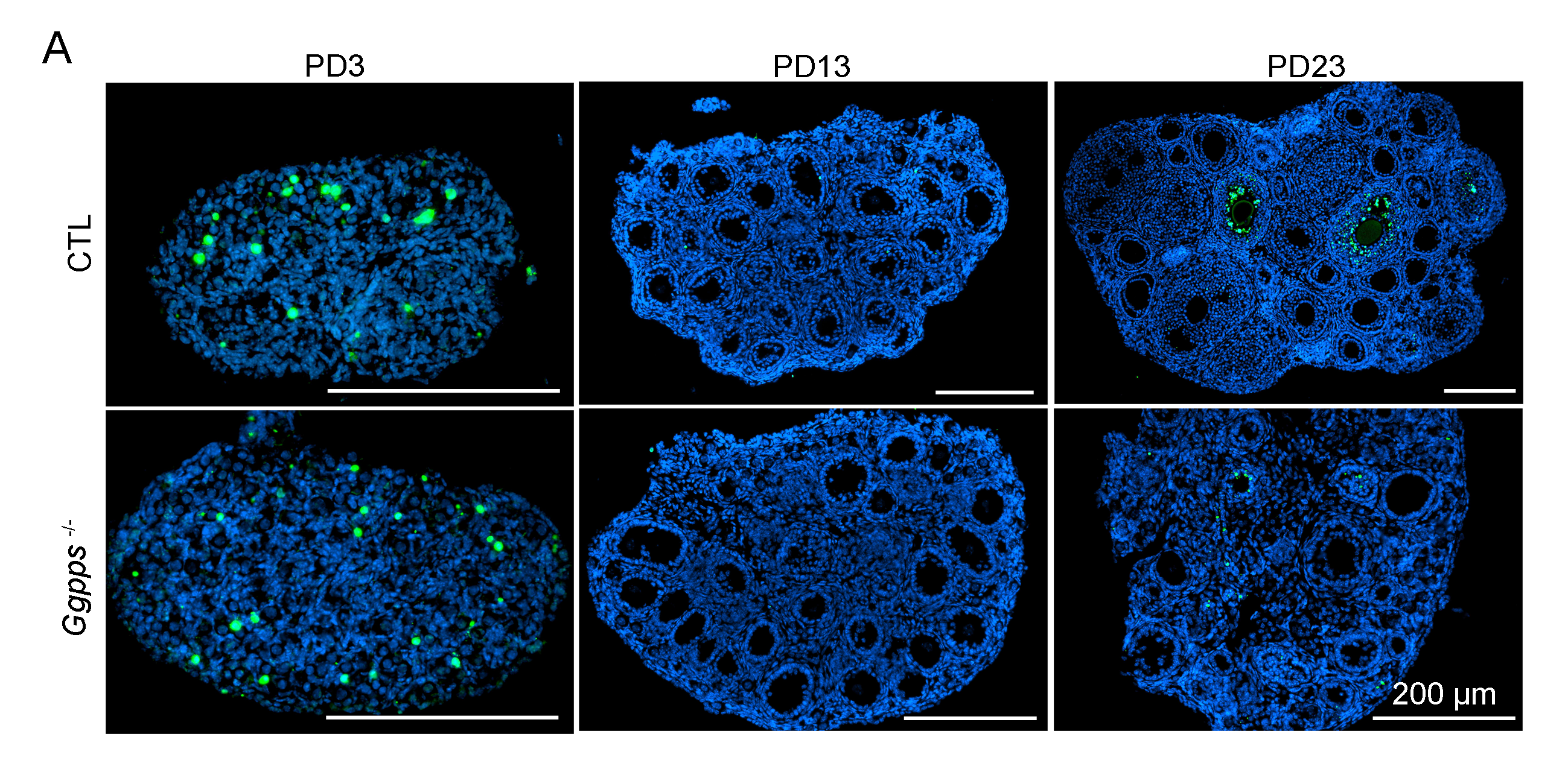

Supplement: S2 Fig — The green dots represent apoptotic cells and DAPI (blue) indicates cell nuclei. Scale bar, 200 μm. (TIF) [file pgen.1006535.s002.tif]

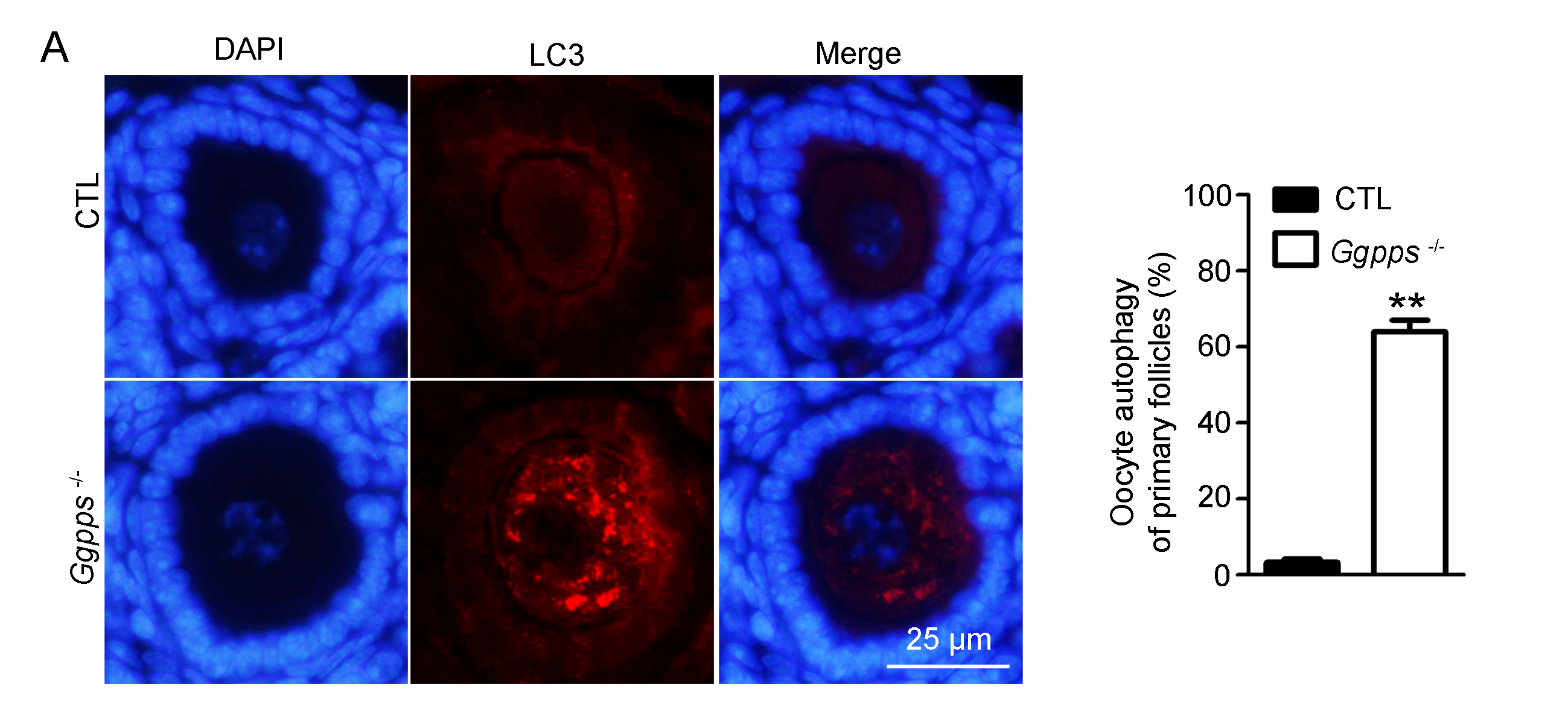

Supplement: S3 Fig — The Red dots represent LC3B and DAPI (blue) indicates cell nuclei. Scale bar, 25 μm. Data were presented as the mean ± SEM. **p<0.01. (TIF) [file pgen.1006535.s003.tif]

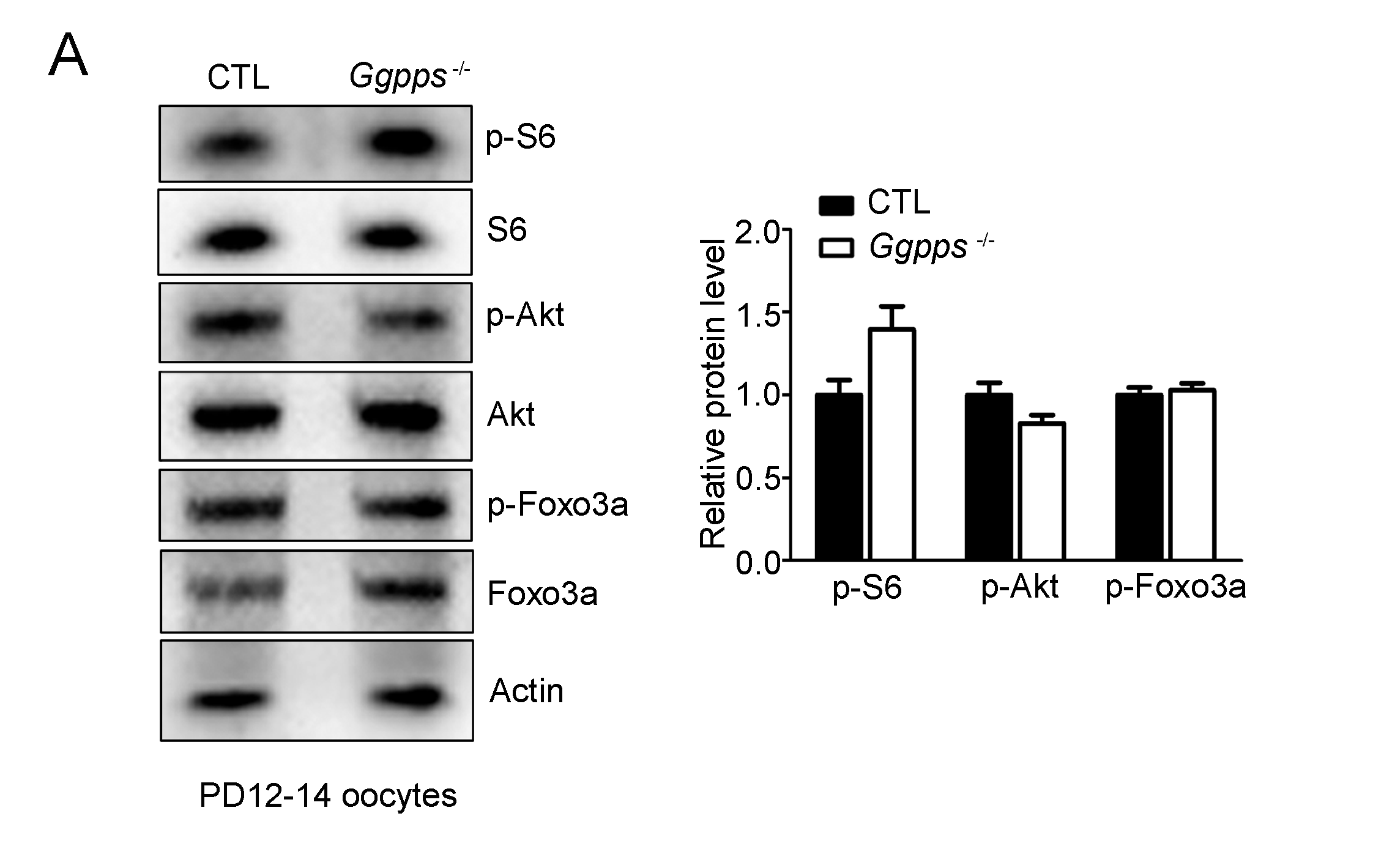

Supplement: S4 Fig — (TIF) [file pgen.1006535.s004.tif]

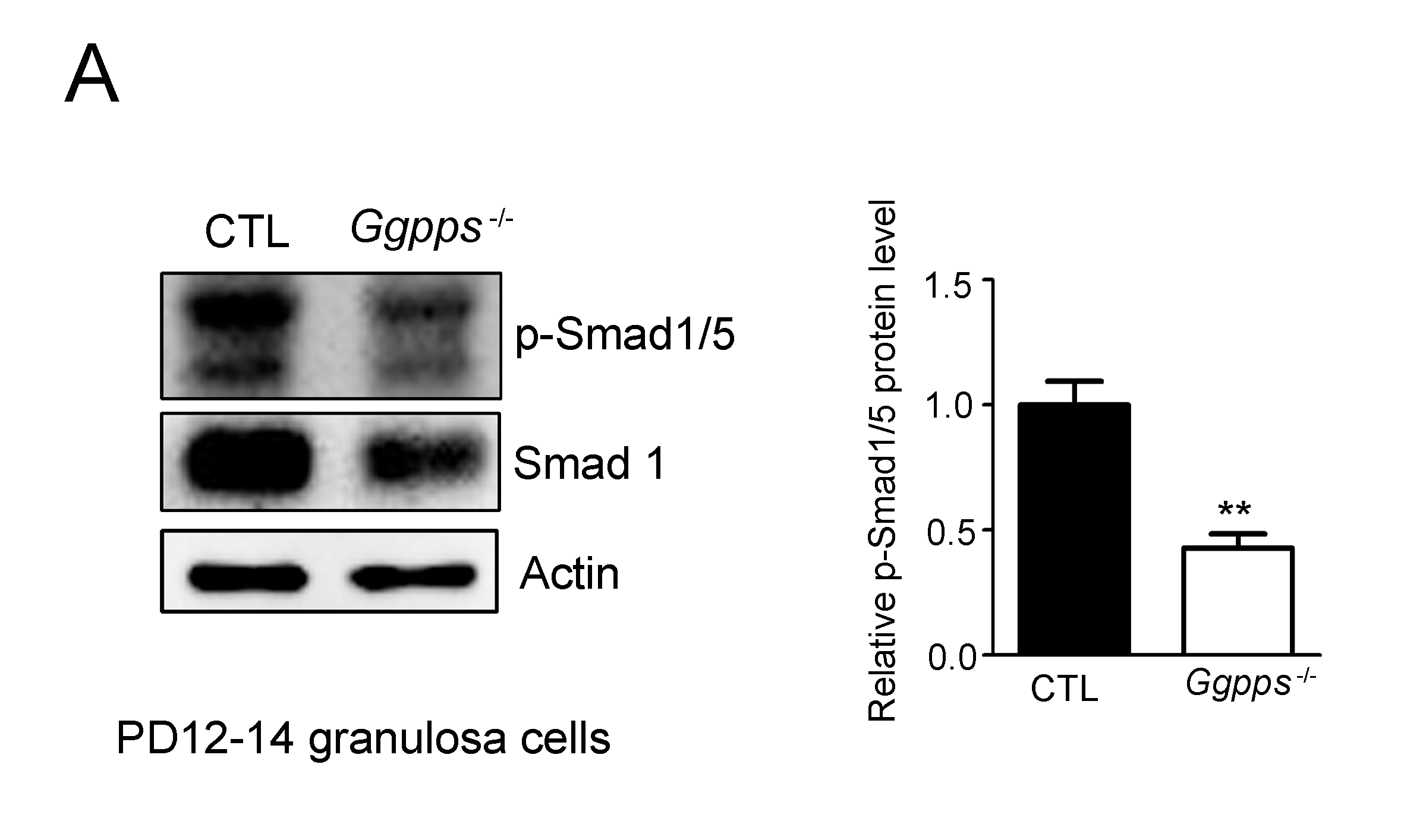

Supplement: S5 Fig — Data were presented as the mean ± SEM. **p<0.01. (TIF) [file pgen.1006535.s005.tif]

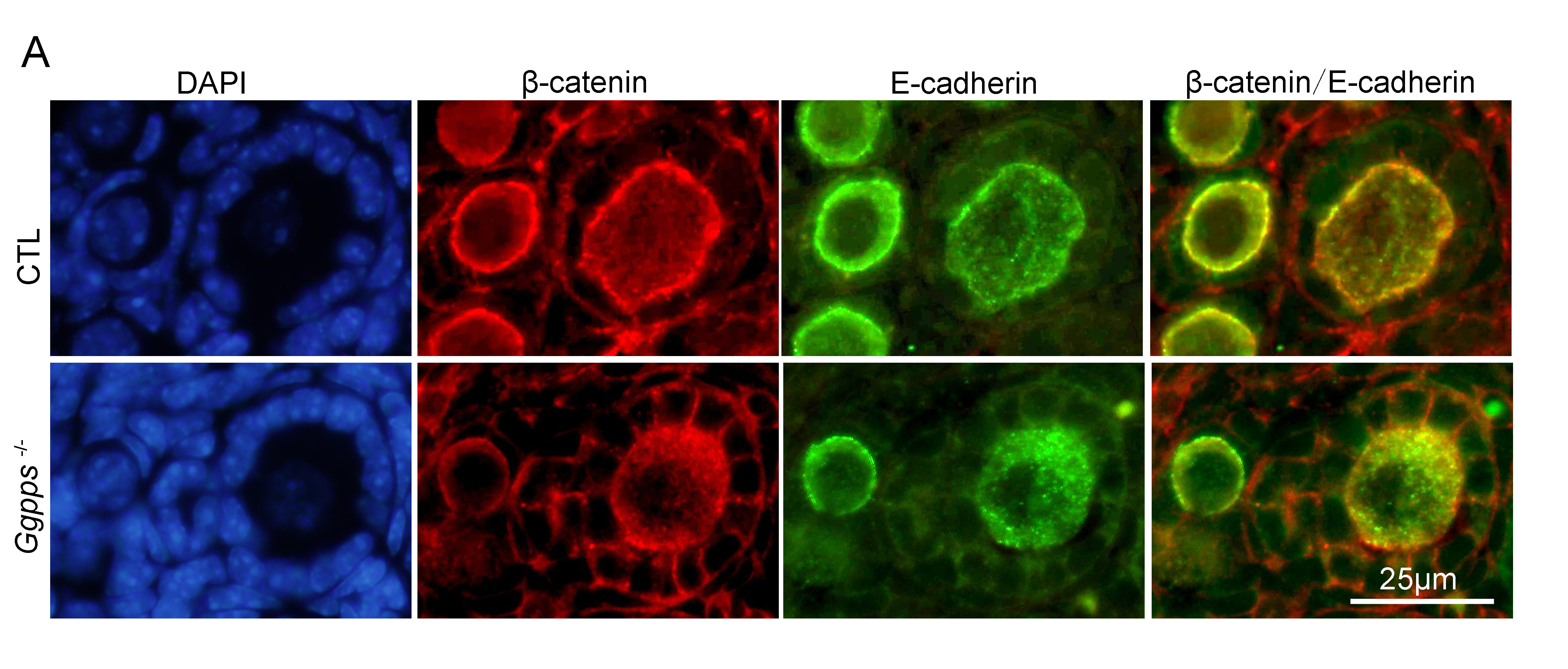

Supplement: S6 Fig — The red represented β-catenin and the green indicates E-cadherin. Scale bar, 25 μm. (TIF) [file pgen.1006535.s006.tif]
