# Supplementary material for: GGPP-Mediated Protein Geranylgeranylation in Oocyte Is Essential for the Establishment of Oocyte-Granulosa Cell Communication and Primary-Secondary Follicle Transition in Mouse Ovary
Source: PLoS Genet. 2017 Jan 10;13(1):e1006535. doi: 10.1371/journal.pgen.1006535 (PMC5224981; doi:10.1371/journal.pgen.1006535)
Supplement: S1 Table — (PDF) [file pgen.1006535.s007.pdf]

Supplemental Table 1. Primer sequences

Mouse:

**Actin-forward**	5’- GGCTGTATTCCCCTCCATCG-3’
**Actin-reverse**	5’- CCAGTTGGTAACAATGCCATGT-3’
**GGPPS-forward**	5’-TTTTGCATACACTCGACACACT-3’
**GGPPS-reverse**	5’-ACCACAGGCCTCAATTTGTTTGT-3’
**Gdf9-forward**	5’- TCTTAGTAGCCTTAGCTCTCAGG-3’
**Gdf9-reverse**	5’- TGTCAGTCCCATCTACAGGCA-3’
**Bmp15-forward**	5’- TCCTTGCTGACGACCCTACAT-3’
**Bmp15-reverse**	5’- TACCTCAGGGGATAGCCTTGG-3’
**Cre-forward**	5’-GCGGTCTGGCAGTAAAAACTATC-3’
**Cre-reverse**	5’-GTGAAACAGCATTGCTGTCACTT-3’
**LoxP-forward**	5’-AATTGTGTGTGGTAGGGGTA-3’
**LoxP-reverse**	5’-AACTTGCTTCAGAACTGAGC-3’
